# A community sensitive mixed-methods protocol for parental reporting of child sexual abuse

**DOI:** 10.1016/j.mex.2026.104034

**Published:** 2026-07-06

**Authors:** Ismiyati, Suminah, Ika Sumiyarsi Sukamto, Argyo Demartoto

**Affiliations:** a Politeknik Kesehatan Kementerian Kesehatan Banten, Banten, Indonesia; b Faculty of Postgraduate School, Universitas Sebelas Maret, Central Java, Indonesia; c Faculty of Medicine, Universitas Sebelas Maret, Central Java, Indonesia; d Faculty of Social and Political Sciences, Universitas Sebelas Maret, Central Java, Indonesia

**Keywords:** Child sexual abuse, Parental reporting, Disclosure delay, Mixed-methods protocol

## Abstract

This article presents a community sensitive mixed methods protocol for studying parental reporting of child sexual abuse. The protocol combines a brief school based cross sectional survey among parents with service linked in-depth interviews involving parents of affected children. It was developed to address a common limitation in child sexual abuse reporting research, namely the separation of community level reporting attitudes from case based family experiences. The protocol uses a low burden four item survey and semi-structured interview guide. The two strands are integrated at the interpretation stage through a narrative joint display that compares normative endorsement of reporting with lived reporting barriers. The protocol specifies the quantitative multistage random sampling procedure, participant flow, qualitative case access involving parents of affected children, thematic coding procedures, and concrete examples of mixed-methods integration to strengthen reproducibility. The protocol is designed to be feasible, ethically sensitive, and reproducible in community and low-resource settings. It may be useful for researchers seeking to study discrepancies between support for formal reporting in principle and the practical difficulties families face when abuse is disclosed.

• Combines community based parent surveying with service linked parental interviewing in one ethically structured design.
• Uses a brief reporting attitudes instrument and a domain based interview guide to generate complementary forms of evidence.
• Provides a replicable workflow for recruitment, safeguarding, qualitative analysis, and mixed methods integration.

## Graphical abstract

**Figure:**
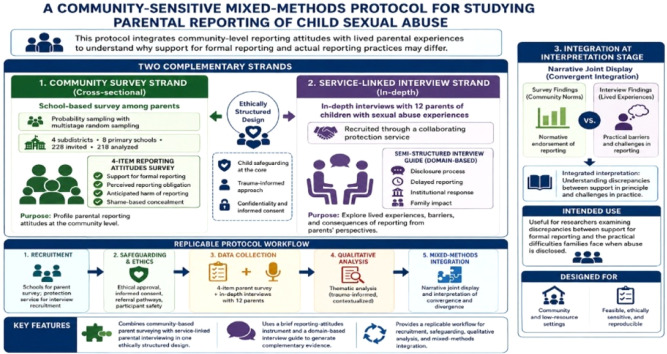

**Specifications table:**

**Table:** 

**Subject area**	Psychology
**More specific subject area**	Child protection psychology; child sexual abuse disclosure and reporting; family response to child sexual abuse; mixed methods research in sensitive community settings
**Name of your method**	Community sensitive mixed methods protocol, Parental reporting of child sexual abuse
**Name and reference of original method**	None
**Resource availability**	None

## Background

Research on child sexual abuse reporting is methodologically challenging because disclosure is often delayed, partial, and shaped by social, emotional, and institutional conditions rather than knowledge alone [[1], [2], [3], [4]]. Community based surveys are useful for assessing general attitudes toward reporting, shame, concealment, and perceived responsibility; however, they may not adequately capture how parents respond when abuse is disclosed within their own family context. Conversely, case based qualitative studies provide detailed accounts of lived experience, but they are less well suited to examining the broader normative environment that shapes disclosure and reporting decisions [5,6]. Taken together, these limitations indicate the need for methodological approaches that can examine both context-specific parental responses and the wider social norms influencing reporting behavior.

The present protocol was developed to address a methodological gap by integrating two forms of evidence that are often examined separately: community level parental attitudes toward reporting and case based parental reporting experiences [5,6]. To achieve this, the protocol employed two recruitment pathways. Schools provided practical access to a broader parent population, whereas a local child protection service offered an ethically safer route for recruiting parents of affected children [7,8]. This dual entry strategy was designed to enhance ethical feasibility, reduce direct exposure risk, and enable comparison between normative support for reporting and the barriers encountered in practice [[7], [8], [9], [10]].

The main methodological contribution of the protocol lies in its ability to identify mismatches between what parents believe should be done in principle and what becomes difficult in practice when abuse is disclosed within a family. This distinction is important because underreporting is often shaped not only by limited knowledge, but also by shame, perceived reputational threat, procedural uncertainty, and concerns about family disruption [1,2,4]. A second contribution is the use of a context sensitive dual entry recruitment strategy. The school based survey provides low burden access to a wider parent population, while service linked recruitment enables ethically safer engagement with parents of affected children. Together, these pathways generate complementary evidence without requiring direct recruitment of children or unscreened outreach to highly vulnerable families. A third contribution is the integration of a brief descriptive instrument, a trauma informed interview procedure, and a predefined workflow for data integration. The protocol was therefore developed not simply as a mixed methods design, but as a replicable workflow for studying reporting processes in sensitive community settings where ethical, cultural, and institutional constraints shape both access and disclosure.

This study presents the protocol as a context sensitive methodological adaptation designed to bridge the gap between community level reporting norms and case based reporting realities in child sexual abuse research. The protocol is useful for researchers examining parental knowledge and attitudes toward reporting, barriers associated with delayed reporting, and the perceived effects of abuse on family functioning within a single design. It may be particularly relevant in low resource or community based settings, where short survey instruments, flexible but structured interviews, and careful procedural safeguards are needed to balance feasibility, confidentiality, and analytical depth [[7], [8], [9], [10], [11], [12]].

## Method details

This study employed a mixed methods design that combined a quantitative cross sectional survey with a qualitative case oriented component to examine parents’ knowledge of and attitudes toward reporting child sexual abuse within the community, as well as the barriers and family impacts experienced by parents of affected children. Mixed methods approaches are appropriate when a research question requires both a broad descriptive profile and an in-depth contextual understanding of processes, meanings, and lived experiences [6]. In this protocol, the survey strand was used to describe parental knowledge of and attitudes toward the formal reporting of child sexual abuse in the wider community, whereas the qualitative strand was used to explore how reporting was understood and negotiated by parents of affected children in real cases. The design followed a complementary logic: the quantitative strand provided a community level reference point, while the qualitative strand explained how reporting decisions were shaped in lived family and service contexts. Integration was undertaken at the interpretation stage through a structured comparison of patterns across the two strands, an approach recommended for identifying convergence, divergence, and complementarity in mixed methods research [5]. To improve reproducibility, the protocol specifies the recruitment pathways, eligibility criteria, quantitative sampling procedure, qualitative coding process, and integration strategy so that the method can be replicated or adapted in comparable community based and low-resource settings.

## Methodological advance over existing practice

The protocol was designed to address a limitation frequently found in studies of child sexual abuse reporting, namely the separation of community level attitude measurement from case based family experience [5,6]. Survey only approaches can identify normative beliefs about disclosure and reporting, but they may not reveal the practical, emotional, and relational barriers that shape parental action when abuse is disclosed in real life contexts [1,2,4]. In contrast, qualitative case studies can illuminate those barriers in depth, but they do not indicate whether such barriers are consistent with or distinct from broader community attitudes. The present protocol addresses this gap by combining a low burden parent survey recruited through schools with service linked interviews involving parents of affected children, followed by planned integration at the interpretation stage [5,6].

This design offers three practical advantages over more conventional single strand approaches. First, it enhances contextual interpretability by allowing descriptive survey patterns to be directly compared with lived reporting experiences [5]. Second, it improves ethical feasibility by separating community recruitment from case based recruitment and by avoiding direct child interviews within the protocol [7,8]. Third, it enhances transferability by using operationally simple components that can be adapted across resource constrained settings: a brief survey instrument, a semi structured interview guide, a trauma informed interviewing workflow, and a predefined integration strategy [[9], [10], [11], [12]]. The protocol should, therefore, be understood as a context sensitive methodological adaptation rather than a routine combination of survey and interview methods [5,6]. (Table 1; Table 2)

**Table 1:** Comparison of the present protocol with commonly used single strand or weakly integrated approaches.

Conventional approach	Main limitation	Improvement introduced by the present protocol	Methodological value
Community survey only	Captures normative beliefs about reporting but does not reveal how reporting barriers operate in real life family situations	Adds service linked qualitative interviews with parents of affected children	Enables comparison between reported attitudes and lived reporting realities
Qualitative case study only	Provides depth on lived experience but lacks a broader community reference point	Adds a school based parent survey to profile reporting related attitudes in the wider community	Strengthens contextual interpretation of case based findings
Direct recruitment of vulnerable families without institutional linkage	May increase ethical risk and reduce participant protection in sensitive contexts	Uses service linked recruitment through an existing protection structure	Improves ethical feasibility and participant safeguarding
Extended survey instruments in sensitive community settings	May increase respondent burden and reduce participation or completion	Uses a brief four item descriptive instrument	Supports low burden implementation in community-based settings
Unstructured integration of quantitative and qualitative strands	Produces parallel findings but weak cross strand interpretation	Uses a predefined narrative joint display	Enhances interpretability by linking patterns across data strands
Child-centered interview protocols in highly sensitive reporting research	May increase ethical and emotional risk when direct child participation is not essential to the research aim	Restricts qualitative participation to parents or caregivers	Improves ethical suitability while preserving access to reporting related experience
Context-free mixed methods designs	May be difficult to adapt in low resource settings with limited institutional access	Uses a context sensitive workflow based on schools and protection services	Improves transferability to similar community and service environments

**Table 2:** Example of narrative joint display for integrating survey patterns and qualitative themes.

Shared analytic domain	Quantitative indicator	Qualitative theme	Integrative interpretation
Formal reporting as prevention	Percentage of parents agreeing that reporting can prevent recurrence	Reporting as protection and prevention	Assesses whether community-level endorsement of reporting is consistent with parents’ lived understanding of reporting as a protective action.
Reporting obligation	Percentage of parents agreeing that all forms of child sexual abuse must be reported	Moral and legal responsibility to report	Examines whether perceived reporting obligation is reinforced or complicated by family, cultural, or institutional concerns.
Anticipated harm of reporting	Percentage of parents agreeing or disagreeing that reporting does more harm than good	Fear of stigma, retaliation, legal burden, or family disruption	Explains why parents may support reporting in principle but hesitate in practice.
Shame-based concealment	Percentage of parents agreeing or disagreeing that abuse should be concealed because of family shame	Shame, silence, and protection of family reputation	Clarifies how cultural and relational meanings of shame may influence disclosure and reporting decisions.
Reporting delay	Not directly measured in the survey	Delayed reporting due to fear, uncertainty, or lack of procedural knowledge	Identifies a qualitative mechanism that may explain the gap between reporting attitudes and actual reporting behavior.
Family impact	Not directly measured in the survey	Emotional, relational, and social consequences for the family	Extends the survey findings by showing how reporting decisions are embedded in broader family consequences.

## Study setting and preparatory procedures

Prior to data collection, the research team established collaborations with two institutional entry points: primary schools for community based survey recruitment and the Protection House for service linked qualitative recruitment. Preparatory work included the development of informed consent materials, confidentiality safeguards, anonymization procedures, and a distress sensitive workflow appropriate for research on a highly sensitive topic.

All field researchers underwent training in trauma informed communication, confidentiality, non leading questioning, and procedures for managing pauses, withdrawals, distress, and referrals. Ethical guidance for violence related research highlights participant safety, privacy, interviewer preparedness, and referral readiness as central methodological requirements, rather than supplementary considerations [7,8]. More recent methodological guidance emphasizes that interviews on sensitive topics benefit from careful preparation, safe interviewing environments, rapport building, reflexivity, and strategies to reduce participant distress and re-traumatization [9,10].

### Replicable implementation workflow

To enhance reproducibility, the protocol can be implemented in the following sequence:

Step 1. Establish institutional collaboration with participating schools and a child protection or victim assistance service.

Step 2. Prepare field materials, including participant information sheets, informed consent forms, anonymization procedures, referral information, and interviewer guidance.

Step 3. Train field researchers in trauma informed communication, confidentiality, distress management, and non leading interviewing techniques.

Step 4. Select the quantitative survey sample using probability sampling with a multistage random sampling technique. In the present implementation, four subdistricts were randomly selected from the target regency to provide geographic representation of the study area.

Step 5. Within each selected subdistrict, classify primary schools by location and randomly select two schools representing central and peripheral school locations.

Step 6. Randomly select of eligible parents or caregivers from each selected primary school and invite them to complete the anonymous four-item survey through school communication channels.

Step 7. Document the quantitative participant flow, including the number of parents invited, questionnaires returned, questionnaires excluded, and the final analytic sample.

Step 8. Screen returned questionnaires for completeness and prepare the quantitative dataset for descriptive analysis.

Step 9. Recruit qualitative participants through the collaborating protection service. In the present implementation, the qualitative component involved parents of children who had experienced sexual abuse and had received assistance from the protection service.

Step 10. Conduct audio-recorded semi-structured interviews with parents only, transcribe them verbatim, de-identify all materials, and import transcripts into qualitative analysis software.

Step 11. Analyze the quantitative and qualitative strands independently, then integrate the findings through a narrative joint display, comparing survey patterns with qualitative themes and explanatory excerpts.

To support reuse of the protocol, the minimum recommended implementation package includes the survey instrument, interview guide, participant information sheet, consent form, anonymization workflow, distress-response procedure, coding framework, and a joint display template. Researchers adapting this method should also prepare a brief implementation log describing any contextual modifications, including changes in the sampling frame, school selection process, protection-service recruitment pathway, interview location, or safeguarding arrangements. Researchers adapting this method to new settings should report any changes to recruitment pathways, interview procedures, or integration logic to ensure that context specific adaptations remain transparent.

## Quantitative component

### Purpose

The quantitative strand was designed to generate a brief descriptive profile of parental knowledge and attitudes toward formal reporting of child sexual abuse. Brief community instruments are useful in sensitive research when the aim is exploratory profiling rather than full psychometric modeling, provided that item clarity and content relevance are established in advance [13].

### Participants and recruitment

The survey was conducted among parents of children recruited through primary schools. This component aimed to capture general parental knowledge and attitudes toward child sexual abuse reporting procedures in the community, rather than the lived experience of specific abuse cases. The quantitative component used a probability sampling approach with a multistage random sampling technique. This sampling strategy was applied to obtain a systematic school-based parent sample representing different subdistrict contexts within the regency. In the first stage, four subdistricts were randomly selected from the target regency to provide geographic representation of the study area. In the second stage, primary schools within each selected subdistrict were grouped by location, and two schools were randomly selected to represent central and peripheral school locations. In the final stage, approximately 28–29 eligible parents or caregivers were randomly selected from each selected primary school and invited to participate in the survey through school communication channels. Eligibility criteria included: (1) being a parent or caregiver of a child enrolled in a participating school, and (2) providing informed consent. Questionnaires were excluded if they contained incomplete responses or could not be used for descriptive analysis. A total of 228 parents were invited to participate in the quantitative survey, and all 228 questionnaires were returned. After data screening, 10 questionnaires were excluded because of incomplete responses. Therefore, the final analytic sample consisted of 218 parents. This participant flow corresponds to a 100% questionnaire return rate and a 95.6% final usable response rate. The survey was administered anonymously; no personally identifiable information was collected, and all responses were kept confidential and used solely for research purposes. School based recruitment can be appropriate for studies focused on community level parental attitudes, as it offers a practical and socially familiar access route. However, recruitment materials should clearly indicate that participation is voluntary and unrelated to the child’s schooling to reduce perceived pressure and safeguard autonomy [8].

### Measure

The survey instrument consisted of four statements designed to assess parents’ knowledge and attitudes toward reporting child sexual abuse to formal authorities. Each item was answered using a binary response format (Agree/Disagree). This brief structure was intended to reduce respondent burden and facilitate administration in community settings where longer questionnaires may not be feasible. The four statements were:

1) “One of the best ways to prevent child sexual abuse from recurring is to report it.”
2) “All forms of child sexual abuse must be reported.”
3) “Reporting child sexual abuse usually does more harm than good.”
4) “Sexual abuse is a shameful act for the family and should be concealed from other people and legal authorities.”

These items were designed to reflect four reporting-related domains frequently identified in the disclosure literature: support for formal reporting, perceived reporting obligation, anticipated harm associated with reporting, and shame-based concealment [[1], [2], [3], [4]].

### Content validation

Content validity was assessed using Aiken’s V with eight experts. Aiken’s V is a well established method for quantifying expert judgment regarding the relevance of items to a target construct and is particularly useful during early stage instrument development or brief tool refinement [13]. In the original implementation, the critical value was 0.81, while Aiken’s V coefficients were 0.875 for item 1, 0.906 for item 2, 0.906 for item 3, and 0.875 for item 4. These values support the adequacy of the items for preliminary descriptive use.

### Quantitative data analysis

Data were analyzed descriptively using frequencies and percentages for each item response. Since the four items were intended to generate a preliminary descriptive profile of reporting related knowledge and attitudes, rather than functioning as a composite scale for inferential modeling, the analysis was restricted to descriptive statistics. This analytical choice is appropriate, as descriptive survey profiles can still make a meaningful contribution to mixed methods research by providing a structured basis for later comparison with qualitative findings [5,6].

## Qualitative component

### Purpose

The qualitative strand was designed to explore how parents of affected children experienced disclosure, reporting decisions, reporting delays, institutional responses, and the perceived impact of abuse on family functioning. Qualitative interviewing is particularly well suited to sensitive topics, as it captures subjective meaning, emotional complexity, and relational processes that are not easily accessible through fixed response instruments [11,12].

### Participants and case access

Qualitative participants were 12 parents of children who had experienced sexual abuse and had received assistance from a protection service. Recruitment through an existing protection service may be preferable to unscreened direct outreach in sensitive settings, as it places participant access within a safeguarding framework and reduces the likelihood of exposing families to unwanted or unprepared contact [7,8]. Participants were selected purposively because the qualitative strand required information-rich cases in which parents had direct experience with disclosure, reporting decisions, institutional responses, delayed reporting, and family consequences following child sexual abuse. Eligible participants were parents or primary caregivers of affected children who had received assistance from the collaborating protection service and were willing to provide informed consent. Parents or caregivers were not approached if participation was considered likely to increase distress, create safety concerns, or interfere with ongoing psychosocial support or legal processes. No direct interviews with child victims were conducted. This decision was made to reduce ethical and emotional risks while still allowing the study to explore reporting related experiences through the perspectives of parents or caregivers.

### Case description and transparency

To improve transparency regarding the qualitative cases, an anonymized overview of case characteristics was provided, including victim age and sex, perpetrator relationship to the victim, and number of perpetrators. Intra community abuse was defined as a subset of extra familial abuse perpetrated by individuals known to the child or family within their immediate social environment, such as peers, neighbors, or other community members. Providing an anonymized case mapping table helps clarify sampling scope and contextual variation without compromising confidentiality.

### Interview guide development

Semi structured interviewing allows researchers to maintain comparability across interviews while remaining responsive to participant pacing, language, and emotional state [11,12]. This flexibility is particularly important in research involving potentially traumatic or highly sensitive experiences [[9], [10], [11]].

The interview guide comprised open ended questions and follow up probes addressing six domains: (1) parental responses to disclosure and reporting; (2) parents’ decision making regarding reporting; (3) perceptions of reporting pathways and legal processes; (4) experiences with institutional and service responses; (5) barriers to delayed reporting; and (6) perceived impacts on family dynamics and functioning.

This domain based structure aligns with the child sexual abuse disclosure literature, which indicates that reporting is shaped by shame, fear of social consequences, uncertainty about procedures, and concern about family disruption [[1], [2], [3], [4]]. Recent evidence syntheses also reinforce the importance of caregiver and parent engagement when child sexual abuse prevention and response are examined in community and education linked contexts [14].

### Interview procedures and safeguarding

Data were collected through voluntary in-depth interviews with 12 parents of children who had experienced sexual abuse. Participants received information on the study objectives and confidentiality procedures, provided written informed consent, and were informed that they could decline to answer any question or withdraw from the interview at any time. Each interview lasted approximately 1.0–1.5 h and was conducted either at the shelter or in the participant’s home, depending on the participant’s preference for privacy and comfort. Given the highly sensitive nature of the topic, particular care was taken to protect participants’ privacy, comfort, and autonomy throughout data collection. Interviews were conducted only with parents or caregivers and did not involve the direct interviewing of children. All qualitative data were de-identified before analysis. This parent-only interview procedure was intended to minimize the possibility of re-traumatization among children while still generating data on disclosure, reporting decisions, institutional responses, and family impacts from the parental perspective.

The interview procedure followed four operational safeguards. First, interviews began with rapport building and less emotionally demanding prompts before progressing to disclosure, reporting decisions, and family consequences. Second, interviewers used open, non leading questions and allowed participants to control the pace and depth of disclosure. Third, if a participant showed signs of distress, the interviewer paused the session, confirmed the participant’s willingness to continue, and, where necessary, provided referral information through the collaborating protection service. Fourth, interviewers recorded field notes after each session to document contextual observations relevant to interpretation, including interruptions, emotional shifts, and deviations from the planned interview flow.

These safeguards were intended to enhance both ethical safety and data quality. Ethical guidance for violence related research emphasizes that participant welfare must take priority over data collection objectives and that referral information should be available when needed [7,8]. More recent methodological guidance similarly recommends careful preparation, safe interview settings, rapport building, open but sensitive questioning, and explicit ethical strategies when interviewing participants about sensitive issues [9,10].

### Qualitative data analysis

In this study, qualitative data were analyzed using thematic analysis. The analytic process involved familiarization with the transcripts, initial coding, grouping codes into candidate themes, iterative refinement of themes in relation to the dataset, and the selection of illustrative excerpts to support each theme. This approach was used to identify salient themes related to barriers to reporting and the perceived impacts of child sexual abuse on families. To support systematic data management and coding, all transcripts were imported into qualitative data analysis software. The coding process began with repeated reading of each transcript to develop familiarity with the data and to identify preliminary meaning units related to disclosure, reporting decisions, institutional response, delayed reporting, stigma, family dynamics, and perceived consequences. Initial codes were generated inductively from the data while remaining informed by the interview domains. Codes with conceptual similarity were grouped into candidate categories, and these categories were subsequently refined into broader themes that represented recurring patterns across cases. Coding was conducted iteratively, with regular peer debriefing among the research team to compare interpretations, address coding inconsistencies, and refine the analytic framework. When differences in coding interpretation occurred, the research team discussed the relevant transcript segments, compared them with the emerging coding framework, and reached agreement through consensus. Analytic decisions, including code merging, code renaming, and theme refinement, were documented to create an audit trail and to strengthen transparency. Candidate themes were then reviewed through repeated comparison of coded extracts with the dataset as a whole to assess their analytic fit, internal coherence, and conceptual clarity. Themes were further refined until consensus was reached within the research team. Thematic analysis is widely used because it offers a flexible yet systematic approach to identifying patterns across qualitative datasets [15,16].

## Integration of quantitative and qualitative strands

The two strands were integrated at the interpretation stage. Interpretation stage integration is appropriate when the strands address related but distinct dimensions of a broader research question and are intended to be compared following independent analysis [5,6]. Integration was undertaken through a narrative joint display that compared descriptive survey patterns with qualitative themes related to disclosure, delayed reporting, institutional responses, and family impact. The joint display was organized around shared analytic domains rather than by data source alone. The shared analytic domains included support for formal reporting, perceived obligation to report, anticipated harm of reporting, shame-based concealment, reporting delay, institutional response, and family impact. For each domain, the survey results were first summarized using frequencies and percentages, and the qualitative findings were then mapped to corresponding themes or explanatory excerpts. This procedure allowed the research team to identify whether the qualitative findings confirmed, expanded, or complicated the descriptive survey patterns. This integration strategy served two methodological purposes. First, it enabled direct comparison between normative endorsement of reporting and the practical barriers described by parents of affected children [5]. Second, it allowed the qualitative strand to explain, contextualize, and complicate descriptive survey patterns rather than merely being presented alongside them [5,6]. For example, if most survey respondents agreed that all forms of child sexual abuse should be reported, this pattern could be compared with qualitative accounts describing why some parents delayed reporting despite recognizing the importance of formal reporting. Similarly, if survey responses showed disagreement with the statement that reporting does more harm than good, qualitative interviews could clarify whether parents nevertheless feared social stigma, family conflict, retaliation, or uncertainty about legal procedures. In this way, the qualitative strand was used not to duplicate the survey findings, but to explain the conditions under which positive reporting attitudes may or may not translate into actual reporting behavior. Through this approach, integration was used not only to combine findings but also to generate a more methodologically informative account of how reporting attitudes and reporting realities interact in sensitive community contexts [5].

## Practical considerations for replication

Researchers adapting this protocol in other settings should consider the local reporting environment, school-parent communication systems, service availability, and cultural meanings attached to shame, disclosure, and legal action. Survey recruitment through schools may be feasible where parent-school communication is well established, but alternative recruitment routes may be required in other contexts. Likewise, qualitative recruitment should ideally occur through a service or safeguarding institution rather than through unscreened direct contact. Researchers should also establish a clear distress management procedure, secure data storage practices, and a context sensitive referral pathway before fieldwork begins [[7], [8], [9], [10]]. To support replication, future users of the protocol should report four minimum implementation details: the quantitative sampling stages and participant flow, participant eligibility and exclusion criteria, the process used to screen and protect qualitative participants, and the specific procedure used to integrate quantitative and qualitative findings. When adaptations are made, these should be described explicitly so that readers can distinguish between the core methodological components of the protocol and context-specific modifications required by local service systems, legal procedures, or cultural norms.

## Method validation

This protocol was validated across four dimensions: content adequacy, operational feasibility, ethical feasibility, and integrative value.

*First,* content adequacy was supported by expert review of the four-item survey instrument using Aiken’s V with eight experts. Item level coefficients ranged from 0.875 to 0.906, exceeding the reported critical value of 0.81, indicating that the items were judged relevant to the intended reporting related domains. These results support the use of the instrument as a brief descriptive tool for profiling parental attitudes toward reporting.

*Second,* operational feasibility was demonstrated through the successful implementation of both strands within the planned field period. The quantitative strand used a multistage random sampling procedure and was completed by 218 parents after the exclusion of 10 incomplete questionnaires from 228 returned questionnaires. This demonstrates that the survey could be administered via existing school-based community communication channels with minimal respondent burden. The qualitative strand was completed with 12 parents of affected children through service-linked recruitment, confirming that ethically sensitive access to affected families was feasible within an institutional safeguarding structure. Interviews, lasting approximately 1–1.5 h, generated analyzable data without requiring direct interviews with children.

*Third,* ethical feasibility was supported by the use of a dual entry recruitment strategy, anonymized survey procedures, informed consent, participant controlled interview pacing, audio recording only with consent, de-identification of qualitative materials, and a distress sensitive interviewing workflow. These elements demonstrate that the protocol can be implemented in a way that preserves participant welfare while still producing interpretable data on a sensitive topic.

*Fourth,* integrative value was supported by the protocol’s ability to generate complementary data that would be less accessible through a single strand design. The survey strand provided a structured profile of community level reporting attitudes, while the qualitative strand clarified how stigma, uncertainty, and family consequences shaped reporting decisions in practice. The use of a narrative joint display enabled these strands to be analytically connected, thereby strengthening interpretation beyond what either strand could provide independently. For this reason, the present protocol should be understood not merely as feasible, but as methodologically useful for identifying discrepancies between normative reporting endorsement and lived reporting barriers.

## Limitations

This method has several limitations. The survey instrument is intentionally brief and is suitable for descriptive profiling rather than full psychometric assessment. The use of binary response options may limit the nuance in parental attitudes. Although the quantitative component used probability sampling with a multistage random sampling technique, the findings should still be interpreted within the specific school-based and community context in which the study was conducted. Accordingly, generalization beyond similar school and community settings should be made cautiously. The qualitative sample was limited to parents already linked to protection services and may therefore underrepresent families who never disclose abuse or never engage with formal support systems.

The protocol also relies on the availability of institutional entry points, particularly school communication systems and a collaborating protection service. In contexts where such structures are weak or inaccessible, recruitment and safeguarding procedures may need to be adapted. Finally, while the protocol strengthens interpretability by integrating community attitudes and case based experiences, it does not eliminate all contextual limitations. Its transferability, therefore, depends on transparent reporting of local legal pathways, service ecology, and cultural meanings attached to shame, disclosure, and family responsibility.

## Ethics statements

This study involved human participants. Ethical approval for this study was granted by the Research Ethics Committee, Faculty of Medicine, Universitas Sebelas Maret (No.173/UN27.06.11/KEP/EC/2024; Protocol ID 158/02/07/2024). Written informed consent was obtained from all participants prior to data collection. Participation was entirely voluntary. Survey responses were collected anonymously, and no personally identifiable information was recorded. For the qualitative component, participants were informed of the study objectives, confidentiality procedures, and their right to decline any question or withdraw from the interview at any time without consequence. Interviews were audio recorded only with participant consent, and all qualitative materials were de-identified prior to analysis. Because the study concerned child sexual abuse, interviews were conducted only with parents or caregivers of affected children, not with children directly, in order to reduce risk and ensure an ethically appropriate procedure for data collection.

## Related research article

None.

## Declaration of generative AI and AI-assisted technologies in the manuscript preparation process

The authors used artificial intelligence tools to improve language clarity during manuscript preparation. The authors reviewed and edited all content and take full responsibility for the accuracy and integrity of the work.

## CRediT authorship contribution statement

**Ismiyati:** Conceptualization, Methodology, Investigation, Resources, Project administration, Writing – original draft. **Suminah:** Formal analysis, Data curation, Validation, Writing – review & editing. **Ika Sumiyarsi Sukamto:** Supervision, Methodology, Writing – review & editing. **Argyo Demartoto:** Supervision, Methodology, Validation, Writing – review & editing.

## Declaration of competing interest

The authors declare that they have no known competing financial interests or personal relationships that could have appeared to influence the work reported in this paper.

## Data Availability

No data was used for the research described in the article.

## References

[1] Alaggia R., Collin-Vézina D., Lateef R. (2019). Facilitators and barriers to child sexual abuse (CSA) disclosures: a research update (2000–2016). Trauma Violence Abuse..

[2] Abdul Latiff M., Fang L., Goh D.A., Tan L.J. (2024). A systematic review of factors associated with disclosure of child sexual abuse. Child Abuse Negl..

[3] Lemaigre C., Taylor E.P., Gittoes C. (2017). Barriers and facilitators to disclosing sexual abuse in childhood and adolescence: a systematic review. Child Abuse Negl..

[4] Mcelvaney R. (2015). Disclosure of child sexual abuse: delays, non-disclosure and partial disclosure. What the research tells us and implications for practice. Child Abuse Rev..

[5] Fetters M.D., Curry L.A., Creswell J.W. (2013). Achieving integration in mixed methods designs-principles and practices. Health. V. Res..

[6] Creswell J.W., Plano Clark V.L. (2017).

[7] WHO (2016). Ethical and safety recommendations for intervention research on violence against women. https://www.who.int/publications/i/item/9789241510189.

[8] WHO (2001). Putting women first: ethical and safety recommendations for research on domestic violence against women. https://www.who.int/publications/i/item/WHO-FCH-GWH-01.1.

[9] Diab J.L., Al-Azzeh D. (2024). Inclusive inquiry: a compassionate journey in trauma-informed qualitative research with GBV survivors from displaced communities. Front. Psychol..

[10] Westland H., Vervoort S., Kars M., Jaarsma T. (2025). Interviewing people on sensitive topics: challenges and strategies. Eur. J. Cardiovasc. Nurs..

[11] DeJonckheere M., Vaughn L.M. (2019). Semistructured interviewing in primary care research: a balance of relationship and rigour. Fam. Med. Community Health..

[12] Kallio H., Pietilä A.M., Johnson M., Kangasniemi M. (2016). Systematic methodological review: developing a framework for a qualitative semi-structured interview guide. J. Adv. Nurs..

[13] Aiken L.R., Three coefficients for analyzing the reliability and validity of ratings, educational and psychological measurement. 45 (1985), 10.1177/0013164485451012.

[14] Russell D.H., Trew S., Harris L., Dickson J., Walsh K., Higgins D.J. (2024). Engaging parents in child-focused child sexual abuse prevention education strategies: a systematic review. Trauma Violence Abuse..

[15] Braun V., Clarke V. (2006). Using thematic analysis in psychology. Qual. Res. Psychol..

[16] Nowell L.S., Norris J.M., White D.E., Moules N.J. (2017). Thematic analysis: striving to meet the trustworthiness criteria. Int. J. Qual. Methods.

